# Seroprevalence and Risk Factors of STIs among Rejected Blood Donors at the National Blood Transfusion Service in Angola

**DOI:** 10.1007/s10461-025-04700-3

**Published:** 2025-04-05

**Authors:** Cruz S. Sebastião, Domingos Jandondo, João Vigário, Felícia António, Joana M. K. Sebastião, Maria L. S. Silva, Victor Pimentel, Ana Abecasis, Euclides Sacomboio, Jocelyne Neto Vasconcelos, Joana Morais

**Affiliations:** 1 Centro Nacional de Investigação Científica (CNIC), Luanda, Angola; 2 Centro de Investigação em Saúde de Angola (CISA)|Instituto Nacional de Investigação em Saúde (INIS), Luanda, Angola; 3 Global Health and Tropical Medicine, Associate Laboratory in Translation and Innovation Towards Global Health, GHTM, LA-REAL, Instituto de Higiene e Medicina Tropical, IHMT, Universidade NOVA de Lisboa, UNL, Rua da Junqueira 100, Lisboa, 1349-008 Portugal; 4 Instituto Nacional de Sangue (INS), Ministério da Saúde, Luanda, Angola; 5 Faculdade de Medicina (FM), Universidade Katyavala Bwila (UKB), Benguela, Angola; 6 Instituto de Ciências da Saúde (ICISA), Universidade Agostinho Neto (UAN), Luanda, Angola

**Keywords:** Sexual transmitted infections, Seroprevalence, Epidemiology, Transfusion medicine, Angola

## Abstract

Sexually transmitted infections (STIs) are a global health concern. Blood donation centres employ comprehensive screening measures to identify donors with STIs, such as HIV, HBV, HCV, and syphilis, which can also transmitted through blood transfusions. Herein, we investigated the seroprevalence of STIs and demographic determinants related to multiple infections among rejected blood donors at the National Blood Transfusion Service (NBTS) in Angola. This was a cross-sectional study conducted with 1885 rejected blood donors serologically screened for anti-HBsAg, anti-HCV, anti-HIV, and anti-TP during pre-donation screening at the NBTS, located in Luanda, the capital city of Angola, between March 2022 to July 2023. Overall, HIV (11.2%), HBsAg (71.7%), HCV (9.30%), and Syphilis (8.80%) were detected. The multiple infection rate was 2.30%. HIV and syphilis were associated with age (*p* < 0.05). HBV was related to age, areas of residence, and occupation (*p* < 0.05). HCV was related to areas of residence and educational level (*p* < 0.05). No relationship was observed between demographic characteristics with multiple infections (*p* > 0.05). Individuals aged over 40 years (OR: 2.48, *p* = 0.393), males (OR: 1.33, *p* = 0.639), non-urbanized regions (OR: 1.18, *p* = 0.594), low educational level (OR: 3.46, *p* = 0.222), and employed (OR: 1.34, *p* = 0.423), were more likely to have multiple infections. Our results demonstrate a high rate of circulation of STIs among blood donation candidates in Luanda. HBV was the main reason for the rejection of candidates. However, nucleic acid-based screening techniques should be considered to ensure better quality screening for occult infections in blood donor candidates.

## Introduction

Blood transfusion plays a vital role in saving countless lives around the world every year, however, it is vital to ensure the safety of transfusions to prevent life-threatening complications and minimise the risk of transfusion-transmitted infections (TTIs), which can also be transmitted through sexual routes. Transfusion in certain regions of sub-Saharan Africa (SSA) is associated with challenges in blood safety due to the high endemicity of TTIs, such as human immunodeficiency virus (HIV), hepatitis B virus (HBV), hepatitis C virus (HCV), and treponema pallidum (TP) [1, 2]. 

The transmission of infections through the blood supply is a significant concern worldwide, mainly in resource-limited countries [3]. Safe donors are most prominent in regions where all donors participate voluntarily [4] as observed in approximately 85% of developed countries compared to around 15% of low- and middle-income countries (LMICs) [5]. Previous studies showed that only around 14% of blood donors in Luanda, the capital city of Angola, are volunteers, compared to 86% of family and replacement donors [6, 7]. 

Given the challenges in ensuring an adequate and secure blood supply, exploring the epidemiological factors that affect blood donors within the community could shed more light on supply and safety concerns. Angola, a country in Southern Africa, faces unique challenges in combating STIs due to limited access to healthcare services, inadequate sexual education, and prevailing social stigma surrounding STIs that contribute to the persistence and spread of these infections [8]. The National Blood Transfusion Service in Angola plays a critical role in ensuring a safe and adequate blood supply for transfusion purposes. However, the screening process for potential blood donors is of paramount importance to safeguard the health of both donors and recipients. One crucial aspect of this screening process is the detection of STIs, including HIV, HBV, HCV, and syphilis, as these infections can be transmitted through blood transfusions, posing significant risks to recipients [9]. This study aimed to determine the seroprevalence of STIs among rejected blood donors at the National Blood Transfusion Service in Angola. By analyzing the presence of TTIs, we strive to comprehensively assess the burden of STIs within the specific population of rejected blood donors and the impact of these TTIs in Angola.

## Materials and Methods

### Study Design and Setting

This was a cross-sectional study performed with 1885 rejected blood donor candidates tested for the TTIs, such as Anti-HIV, Anti-HBsAg, Anti-HCV, and Anti-TP, between March 2022 to July 2023 at the National Blood Transfusion Service in Angola, located in Luanda, the capital city of Angola. The study was carried out under the responsibility of the Angolan Health Research Center (CISA)| National Institute for Health Research (INIS). The CISA (http://www.cisacaxito.org/pt/cisa/) is an Angolan-specialized regional research and development institution that belongs to INIS (http://www.inis.ao/index.php/institucional/o-instituto), a body supervised by the Angolan Ministry of Health (MoH) that promotes Health Research, whose main objective is to generate, develop and disseminate scientific, technological, and strategic knowledge about health and its determinants, for strengthening public health policies and improving the national health system in Angola. The study protocol received ethical approval from the National Ethics Committee of the Ministry of Health of Angola (nr.39/C.E/2021) and by the National Blood Transfusion Service in Angola (nr.128/GDG/INIS/2022). Moreover, informed consent was secured from all participants before being enrolled in the study. The information obtained was fully anonymized and kept confidential on the responsibility of the CISA|INIS main researcher assigned to this study.

## Data, Sample Collection, and Laboratory Procedure

To collect sociodemographic data from the participants, the research team utilized a demographic questionnaire validated by the Ministry of Health and used by all blood transfusion centres in the country. This questionnaire encompassed various aspects such as age, sex, area of residence, educational background, and marital status. Furthermore, as part of the study protocol, each participant graciously contributed around 5 mL of intravenous whole blood, which was collected into tubes containing ethylenediaminetetraacetic acid (EDTA). The collected samples underwent screening for Anti-HIV (Abbott, USA), Anti-HBsAg (Abbott, USA), Anti-HCV (Abbott, USA), and Anti-TP (Abbott, USA) using the ARCHITECT Plus i2000SR (Abbott, USA), following the instructions provided by the manufacturers. All patients reactive to these pathogens were excluded from blood donation and included in this study to understand the main causes of rejection of blood donation, considering serological status.

### Statistical Analysis

The collected data was analyzed using IBM SPSS Statistics version 26 (SPSS v26). Descriptive analyses were performed, presenting frequencies and percentages. For data that followed a normal distribution, the mean, standard deviation (SD), and the independent samples t-test were reported, such as the age of the participants. To investigate associations between qualitative variables, both the Chi-square (X^2^) test to test the relationship with individual infections and logistic regression to test for the relationship with any of the infections were utilized. Furthermore, the odds ratio (OR) along with 95% confidence intervals (CIs) was calculated. Throughout the study, two-tailed p-values were reported, and statistical significance was determined when *p* < 0.05.

## Results

### Seroprevalence of Sexually Transmitted Infections and Risk Factors Related to Multiple Infections

Table 1 provides an overview of the sociodemographic factors that influence the prevalence of STIs and the associated risk factors for multiple infections. The study included participants aged between 18 and 63 years, with an average age of 30.8 ± 9.07 years. Participants aged between 20 and 40 years (81.5%, 1537/1885), male (91.2%, 1719/1885), living in urbanized areas (58.5%, 1102/1885), with a low educational level (92.7%, 1747/1885), employed (71.9%, 1355/1885), and unmarried (96.0%, 1809/1885), were the predominant population in this study. Overall, anti-HIV (11.2%, 211/1885), anti-HBsAg (71.7%, 1352/1885), anti-HCV (9.30%, 179/1885), and anti-TP (8.80%, 165/1885) were detected. The multiple infection rate was 2.30% (44/1885) whether for HIV/HBV (1.10%), HIV/HCV (0.60%), HIV/Syphilis (1.20%), HBV/HCV (8.0%), HBV/Syphilis (5.50%), and HCV/Syphilis (1.80%) (data not shown). The mean ages of the individuals infected with HIV (33.8±8.90, years, *p* < 0.001), HCV (30.8±9.03, years, *p* = 0.555), and Syphilis (30.1±8.53, years, *p* < 0.001) were higher than those of the uninfected. On the other hand, HBV-infected individuals (29.4±8.16, *p* < 0.001) had lower average ages compared to uninfected individuals.

**Table 1:** Seroprevalence of sexually transmitted infections and risk factors related to coinfection among rejected blood donors in Angola

Independent variables	*N* (%)	Anti-HIV			Anti-HBsAg			Anti-HCV		
		No (%)	Yes (%)	*p*-value	No (%)	Yes (%)	*p*-value	No (%)	Yes (%)	*p*-value
Overall	1885 (100)	1674 (88.8)	211 (11.2)		533 (28.3)	1352 (71.7)		1709 (90.7)	176 (9.30)	
Age (years), mean ± SD	30.8 ± 9.07	30.4 ± 9.02	33.8 ± 8.90	**< 0.001**	34.4 ± 10.2	29.4 ± 8.16	**< 0.011**	30.8 ± 9.03	31.2 ± 9.42	0.555
<20yrs	74 (3.90)	70 (4.20)	4 (1.90)	**0.005**	17 (3.20)	57 (4.20)	**< 0.001**	67 (3.90)	7 (4.00)	0.104
20-40yrs	1537 (81.5)	1375 (82.1)	162 (76.8)		380 (71.3)	1157 (85.6)		1403 (82.1)	134 (76.1)	
>40yrs	274 (14.5)	229 (13.7)	45 (21.3)		136 (25.5)	138 (10.2)		239 (14.0)	35 (19.9)	
Gender										
Female	166 (8.80)	144 (8.60)	22 (10.4)	0.378	56 (10.5)	110 (8.10)	0.102	151 (8.80)	15 (8.50)	0.889
Male	1719 (91.2)	1530 (91.4)	189 (89.6)		477 (89.5)	1242 (91.9)		1558 (91.2)	161 (91.5)	
Residence area										
Non-urban	783 (41.5)	685 (40.9)	98 (46.4)	0.125	253 (47.5)	530 (39.2)	**0.001**	691 (40.4)	92 (52.3)	**0.002**
Urban	1102 (58.5)	989 (59.1)	113 (53.6)		280 (52.5)	822 (60.8)		1018 (59.6)	84 (47.7)	
Educational level										
Low	1747 (92.7)	1548 (92.5)	199 (94.3)	0.334	503 (94.4)	1244 (92.0)	0.077	1576 (92.2)	171 (97.2)	**0.017**
High	138 (7.30)	126 (7.50)	12 (5.70)		30 (5.60)	108 (8.00)		133 (7.80)	5 (2.80)	
Occupation										
Unemployed	530 (28.1)	446 (27.8)	64 (30.3)	0.448	169 (31.7)	361 (26.7)	**0.029**	470 (27.5)	60 (34.1)	0.064
Employed	1355 (71.9)	1208 (72.2)	147 (69.7)		364 (68.3)	991 (73.3)		1239 (72.5)	116 (65.9)	
Marital status										
Unmarried	1809 (96.0)	1604 (95.8)	205 (97.2)	0.352	514 (96.4)	1295 (95.8)	0.517	1637 (95.8)	172 (97.7)	0.213
Married	76 (4.00)	70 (4.20)	6 (2.80)		19 (3.60)	57 (4.20)		72 (4.20)	4 (2.30)	

Independent variables	*N* (%)	Anti-TP			Multiple infections^a^			Univariate analysis	
		No (%)	Yes (%)	*p*-value	No (%)	Yes (%)	*p*-value	OR (95% CI)	*p*-value
Overall	1885 (100)	1720 (91.2)	165 (8.80)		1841 (97.7)	44 (2.30)			
Age (years), mean ± SD	30.8 ± 9.07	30.1 ± 8.53	38.2 ± 11.0	**< 0.001**	30.8 ± 9.07	30.8 ± 9.18	0.998		
<20yrs	74 (3.90)	69 (4.00)	5 (3.00)	**< 0.001**	73 (4.00)	1 (2.30)	0.472	1.00	
20-40yrs	1537 (81.5)	1441 (83.8)	96 (58.2)		1503 (81.6)	34 (77.3)		1.65 (0.22–12.2)	0.623
>40yrs	274 (14.5)	210 (12.2)	64 (38.8)		265 (14.4)	9 (20.5)		2.48 (0.31–19.9)	0.393
Gender									
Female	166 (8.80)	146 (8.50)	20 (12.1)	0.116	163 (8.90)	3 (6.80)	0.638	1.00	
Male	1719 (91.2)	1574 (91.5)	145 (87.9)		1678 (91.1)	41 (93.2)		1.33 (0.41–4.33)	0.639
Residence area									
Non-urban	783 (41.5)	715 (41.6)	68 (41.2)	0.929	763 (41.4)	20 (45.5)	0.594	1.18 (0.65–2.15)	0.594
Urban	1102 (58.5)	1005 (58.4)	97 (58.8)		1078 (58.6)	24 (54.5)		1.00	
Educational level									
Low	1747 (92.7)	1596 (92.8)	151 (91.5)	0.548	1704 (92.6)	43 (97.7)	0.193	3.46 (0.47–25.3)	0.222
High	138 (7.30)	124 (7.20)	14 (8.50)		137 (7.40)	1 (2.30)		1.00	
Occupation									
Unemployed	530 (28.1)	482 (29.0)	48 (29.1)	0.771	520 (28.2)	10 (22.7)	0.421	1.00	
Employed	1355 (71.9)	1238 (72.0)	117 (70.9)		1321 (71.8)	34 (77.3)		1.34 (0.66–2.73)	0.423
Marital status									
Unmarried	1809 (96.0)	1652 (96.0)	157 (95.2)	0.577	1765 (95.9)	44 (100)	0.169	1.00	
Married	76 (4.00)	68 (4.00)	8 (4.80)		76 (4.10)	0 (0.00)		0.00 (0.00–0.00)	0.997

Note: Bold numbers mean that results were statistically significant for the Independent Samples T-test or Chi-square (X2) test (*p* < 0.05)

Abbreviations: OR - odds ratio; CI - confidence interval

^a^HIV/HBV, HIV/HCV, HIV/Syphilis, HBV/HCV, HBV/Syphilis, or HCV/Syphilis

The age was significantly related to HIV, HBV, and Syphilis (*p* < 0.05). Specifically, HBV infection was statistically related to age group (20–40 age group most affected with 85.6%, *p* < 0.001), areas of residence (the urbanized area most affected with 60.8%, *p* = 0.001), and occupation (employed individuals most affected with 73.3%, *p* = 0.029), while HCV infection was statistically related to areas of residence (non-urbanized areas most affected with 52.3%, *p* = 0.002) and educational level (low educational level most affected with 97.2%, *p* = 0.017).

Based on the analysis conducted, no significant associations were found between any of the demographic characteristics examined and the occurrence of multiple infections (*p* > 0.05). However, it is worth noting that individuals aged over 40 [OR: 2.48 (CI: 0.31–19.9), *p* = 0.393], males [OR: 1.33 (CI: 0.41–4.33), *p* = 0.639], those residing in non-urbanized areas [OR: 1.18 (CI: 0.65–2.15), *p* = 0.594], individuals with a low educational level [OR: 3.46 (CI: 0.47–25.3), *p* = 0.222], and employed individuals [OR: 1.34 (CI: 0.66–2.73 ), *p* = 0.423], appeared to have a slightly higher likelihood of having multiple infections compared to other groups.

## Discussion

STIs are a public health concern worldwide, and their transmission through blood transfusions can have serious consequences. Sub-Saharan Africa exhibits a significant prevalence of TTIs, including HBV (6.1%), HCV (1.6%), HIV (0.4–28.3%), and syphilis (4–5%) [10]. Herein, 11.2% of donors were found to have anti-HIV antibodies, indicating exposure to HIV, 71.7% were HBsAg-positive, which suggests exposure to HBV, 9.30% had anti-HCV antibodies, indicating exposure to HCV, and 8.80% tested positive for anti-TP, indicating exposure to *Treponema pallidum*, the bacterium responsible for causing syphilis. Studies conducted in other African countries also observed a high rate of HBV infection as observed in this studied population. HBV infection has been at an intermediate or high level of endemicity in LMICs in the last five decades even with the implementation of vaccination programs [11]. The likely reason for the high prevalence of anti-HBsAg might be, on the one hand, the higher infectivity of HBV when compared to HIV, HCV, and syphilis and, on the other hand, the weak community awareness about HBV transmission and prevention, showing that vaccination and awareness programs must be urgently reviewed to reduce the high circulation of HBV, especially in employed young people aged 20–40 year and from urbanized areas (Table 1), who were statistically related to HBV infection (*p* < 0.05). Future studies on the impact of asymptomatic hepatitis with anti-HBsAg positivity on liver function and general clinical outcomes in the young population should be conducted in Angola. Furthermore, molecular studies to determine biological/non-biological factors and HBV strains must be urgently carried out in different populations in Angola. Based on a study carried out by Nebenzahl et al. with individuals attending anonymous testing in Luanda, in 2013, we show an increase in rates of HIV (8.8–11.2%), HBV (9.30–71.7%), HCV (8.1–9.3%), and reduction for syphilis (10–8.8%), from 2013 to 2023 [12]. It is worth noting that the multiple infection rates in the studied population were relatively low, at 2.3%, meaning that a small proportion of participants had more than one of the detected infections simultaneously. The coinfection (HIV/HBV, HIV/HCV, HIV/Syphilis, HBV/HCV, HBV/Syphilis, and HCV/Syphilis) were found to have a statistically significant relationship with a p-value of less than 0.001 (Table 2), suggesting shared risk factors or biological interactions that contribute to their co-occurrence. Similar rates of coinfection such as 2.3% for HIV/HBV, 0.9% for HIV/HCV, and 0.9% (4/431) for HCV/HBV, were previously recorded in the general population from Luanda [12]. These similarities to studies previously carried out with the local population show that the epidemiological, clinical, and behavioural profile as well as clinical outcomes of the population with multiple infections need to be further explored in future studies.

The findings from Table 1 reveal several important trends. Age appears to be a significant factor influencing STI prevalence, with higher rates observed among younger individuals (ages up to 40 years). Our results align with previous research indicating that adolescents and young adults are more vulnerable to STIs in LMICs [13, 14]. Also, Shannon et al., reported that adolescents are responsible for approximately half of new STIs in the United States each year [15]. Risk behaviours such as increased unprotected sex or multiple sexual partners, limited access to comprehensive sexual health education, and weak immunity might explain elevated rates of STIs among these young populations. However, implementing comprehensive sex education programs as well as regular testing in schools and communities could help reduce the rate of STIs, especially in the young population from Angola. Indeed, a study carried out in Huambo, one of the provinces of Angola, showed a significant lack of knowledge about human biology and sexuality among young students in that region [16]. Gender disparities are evident in this studied population (91.1% of male donors), with higher STI prevalence observed among male donors in addition to being 1.33 times more likely to present multiple infections, highlighting the significance of considering gender-specific factors when designing prevention and treatment strategies (Table 1). Women are noticeably underrepresented among blood donors in many studies conducted in different parts of Angola, although they represent more than 50% of the general population [17]. In many studies conducted in Africa, it has been consistently observed that there is a higher participation of males in blood donation programs compared to women [2]. In previous studies conducted in Nigeria (98.7%) [18], Ghana (92.2%) [19], Mauritania (95%) [20], and Mali (88.8%) [21], men have also predominated blood donations, as observed in the present study. A study carried out by Peliganga et al. in Bié, one of the southern provinces of Angola, also observed that men were predominant at 73.4%, suggesting more participation by women from the south in blood donation activities compared to women in the country’s capital (Table 1) [22]. Obstetric factors such as menstrual cycle, pregnancy, and breastfeeding have been plausible explanations attributed to low adherence of women to blood donation in many LMICs. Interestingly, this pattern appears to differ slightly from previous studies in some developed European countries, such as Austria where women blood donors represented 40%, France (49.7%), Norway (50%) and, Great Britain (55%) [2]. 

The sociodemographic factors examined in this study shed light on the complex interplay between STI prevalence and numerous social determinants. For instance, education level, occupation, and residence area emerge as crucial factors. Lower educational level was associated with higher STI rates, specifically, more than 90% for all HIV, HBV, HCV and syphilis infections, emphasizing the importance of promoting accessible and accurate sexual health education. Several studies conducted on SSA indicate a potential correlation between higher levels of education and a reduced likelihood of infection among individuals who donate blood [23, 24], as observed in this study, where donors with a low educational level were 3.46 times more likely to be infected and present multiple infections. Previous studies have documented higher rates of STIs and other communicable diseases in Luanda, partly due to the high concentration of risk groups such as sex workers and injecting drug users [25–30]. Likewise, studies carried out in Ethiopia and Pakistan also showed that the majority of blood donation candidates with positive serology came from urbanized regions [31, 32]. Although not statistically significant (*p* > 0.05), more than 95% of STIs and 100% of multiple infections were observed in unmarried subjects, indicating that this group may serve as a potential source of STI dissemination (Table 1). The reasons why unmarried people constitute an important factor in the spread of STIs in Luanda need to be further studied. Unmarried blood donors have also been reported in Ethiopia [31], Brazil [33], and Pakistan [32] as being those with the highest risk and STI positivity rate.

This study has important limitations. Firstly, our study’s cross-sectional design captures a picture of the seroprevalence rates and risk factors at a specific point in time. Longitudinal studies would provide a more comprehensive understanding of the trends and changes in STI prevalence and associated risk factors over time. Secondly, our study focuses specifically on rejected blood donors, which might not be representative of the general population. As a result, the prevalence rates and risk factors identified in this study may not accurately reflect the broader status of STIs and associated factors. Thirdly, our study is specific to the National Blood Transfusion Service in Angola and may not be directly applicable to other regions or countries. Factors such as local epidemiology, cultural practices, and healthcare infrastructure can influence the prevalence rates and risk factors associated with STIs. Finally, the accuracy of STI testing methods used in our study is crucial for obtaining reliable results. However, variations in testing techniques, sensitivity, and specificity of the diagnostic assays employed can introduce potential limitations and impact the accuracy of the seroprevalence rates reported when compared to the nucleic acid-based screening techniques. Despite these limitations, our study has significant implications for public health since understanding the prevalence and patterns of STIs among individuals who are ineligible to donate blood, we can address several key areas such as (i) donor screening and blood safety, (ii) STI prevention and education, (iii) public health policy and resource allocation, and (iv) STIs surveillance and monitoring. Therefore, our findings may contribute to the establishment of a robust STI surveillance system so that public health authorities can track STI trends over time, guide the implementation of timely interventions, and assess the effectiveness of prevention measures existing in Angola.

## Conclusion

In summary, our study provided valuable insights into the prevalence rates, coinfection patterns, and associated risk factors of STIs among rejected blood donors. Our findings can be used to improve donor screening protocols as well as enhance blood safety, helping minimize the risk of transmitting STIs through blood transfusions and protect the health of recipients. Moreover, our findings can inform public health policy development and resource allocation prioritizing interventions based on the specific STIs that are most prevalent among rejected blood donors ensuring more efficient use of healthcare resources and implementing targeted efforts to combat these infections in Angola.

## Data Availability

All data generated or analysed during this study are included in this published article.
